# Metabolism of Cholesterol and Bile Acids by the Gut Microbiota

**DOI:** 10.3390/pathogens3010014

**Published:** 2013-12-30

**Authors:** Philippe Gérard

**Affiliations:** 1 INRA, UMR1319 Micalis, Jouy-en-Josas F-78350, France; E-Mail: Philippe.Gerard@jouy.inra.fr; Tel.: +33-134-652-428; Fax: +33-134-652-462; 2 AgroParisTech, UMR Micalis, Jouy-en-Josas F-78350, France

**Keywords:** coprostanol, secondary bile acids, deconjugation, epimerization

## Abstract

The human gastro-intestinal tract hosts a complex and diverse microbial community, whose collective genetic coding capacity vastly exceeds that of the human genome. As a consequence, the gut microbiota produces metabolites from a large range of molecules that host's enzymes are not able to convert. Among these molecules, two main classes of steroids, cholesterol and bile acids, denote two different examples of bacterial metabolism in the gut. Therefore, cholesterol is mainly converted into coprostanol, a non absorbable sterol which is excreted in the feces. Moreover, this conversion occurs in a part of the human population only. Conversely, the primary bile acids (cholic and chenodeoxycholic acids) are converted to over twenty different secondary bile acid metabolites by the gut microbiota. The main bile salt conversions, which appear in the gut of the whole human population, include deconjugation, oxidation and epimerization of hydroxyl groups at C3, C7 and C12, 7-dehydroxylation, esterification and desulfatation. If the metabolisms of cholesterol and bile acids by the gut microbiota are known for decades, their consequences on human health and disease are poorly understood and only start to be considered.

## 1. Introduction

If fetuses are sterile in uteri, bacteria from the mother and the surrounding environment colonise the infant’s gut rapidly after birth. This microbiota changes during the first years of life, under the control of different factors including the effects of the microbiota itself, developmental changes in the gut environment, the host genotype, and transition to an adult diet [1]. The final composition of the microbiota is therefore unique and specific of each individual but the factors guiding this feature are still a matter of debate. Adult humans are colonized by microbes from nine divisions (deep evolutionary lineages) of Bacteria and at least one division of Archaea. This represents only a small fraction of the more than 70 bacterial and 13 archaeal divisions known in the biosphere. Moreover, three bacterial divisions, the Firmicutes (gram-positive), Bacteroidetes (gram-negative) and Actinobacteria (gram-positive) dominate the adult human gut microbiota [2].

It is now recognized that the communities of microbes in our gut function as an organ with many metabolic, immunologic and endocrine-like actions that influence human health [3]. Using molecular techniques, it is now estimated that the human gastrointestinal tract harbors approximately 10^14^ microorganisms (10 times more cells than the whole human body) and that this community is composed of 500 to 1000 distinct bacterial species [4]. Moreover, the microbiome contains at least 100 times as many genes as the human genome [5], most of them serving human physiological functions. As examples, the microbiota ferments otherwise indigestible food components, synthesizes vitamins and other essential micronutrients, metabolizes dietary toxins and carcinogens, assures the maturation of the immune system, affects the growth and differenciation of enterocytes, regulates intestinal angiogenesis, protects against enteric pathogens, and converts steroids [6]. Steroids are a family of organic compounds consisting of a five-ring perhydrocyclopentanophenanthrene nucleus. Different classes of steroids, including cholesterol, bile acids and steroid hormones are exposed to the gut microbiota, and subsequently undergo microbial metabolism leading to various metabolites. In the present review, the microbial metabolism in the gut of two classes of steroids is described: Cholesterol originating from the diet or synthesized de novo in the liver and other tissues; Bile acids synthesized from cholesterol in the liver and excreted via the biliary tract.

## 2. Metabolism of Cholesterol by the Gut Microbiota

Every day, up to 1 g of cholesterol enters the colon. Indeed, despite huge inter-individual variations, only half of the dietary cholesterol is absorbed on average, primarily in the duodenum and proximal jejunum. This unabsorbed dietary cholesterol represents around 200 mg/day and is added to the biliary cholesterol secretion, the main source of cholesterol in the lumen, and to cholesterol of cells sloughed from the intestinal epithelium. More recently, direct non-biliary excretion of plasma-derived cholesterol into the intestinal lumen via a pathway termed transintestinal cholesterol efflux (TICE) has been described [7]. Although its contribution to cholesterol excretion is unclear in humans, TICE accounts for up to 70% of fecal neutral sterol excretion in mice. All cholesterol arriving in the large intestine can be metabolized by the colonic bacteria. Indeed, intestinal cholesterol conversion was established as far back as the 1930s [8] and it was subsequently showed that intestinal microbiota was responsible as germfree rats only excreted unmodified cholesterol [9]. Therefore, it was revealed that cholesterol is reduced to coprostanol and minor amounts of coprostanone by the intestinal microbiota. In humans, this microbial conversion of cholesterol started during the second half of the first year of life [10]. Several studies have also reported that the rate of microbial cholesterol-to-coprostanol conversion in human populations was bimodal, with a majority of high converters (almost complete cholesterol conversion) and a minority of low or inefficient converters (coprostanol content representing less than one-third of the fecal neutral sterols content) [11,12]. These conversion patterns were found equally distributed with respect to sex and were independent of age, and the efficiency of cholesterol conversion results mainly from the abundance of cholesterol-reducing bacteria. In a study including fifteen human volunteers, it was established that the level of cholesterol-reducing bacteria must be at least 10^6^ cells/g (wet weight) of stool to efficiently convert cholesterol in the human gut, while a population containing more than 10^8^ cells/g (wet weight) of stool leads to nearly complete conversion. Moreover, a correlation was detected between the overall structure of the fecal microbial community and the cholesterol-reducing activity in the human gut [11].

**Figure 1:**
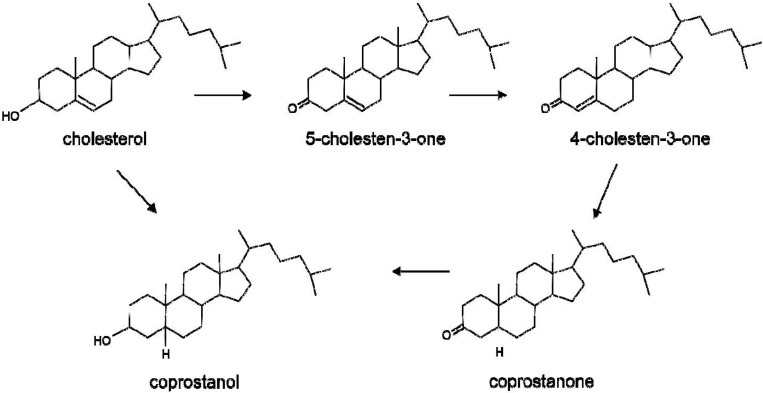
Direct and indirect pathways for the conversion of cholesterol to coprostanol by the gut microbiota.

Two major pathways have been proposed for the conversion of cholesterol to coprostanol [13] (Figure 1). The first pathway involves direct reduction of the 5–6 double bond. The second pathway starts with the oxidation of the 3β-hydroxy group and isomerization of the double bond to yield 4-cholesten-3-one, which undergoes two reductions to form coprostanone and then coprostanol. This second pathway is supported by the presence of coprostanone in human feces and by the reduction of intermediate products to coprostanol by fecal samples. Nevertheless, both pathways may coexist in the human gut.

**Figure 2:**
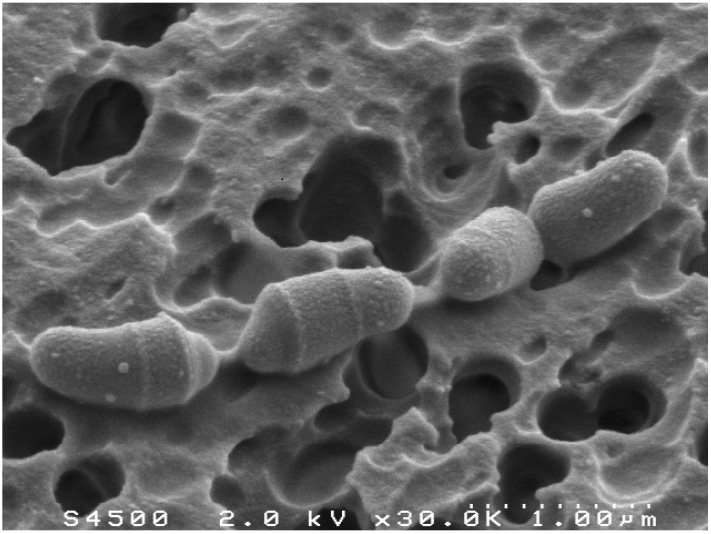
Scanning electron micrograph of *Bacteroides* sp. strain D8, the first cholesterol-reducing bacterium isolated from human feces.

Numerous attempts have been made in order to isolate cholesterol-reducing bacteria. A first cholesterol-reducing strain has been isolated from rat cecal contents, using a cholesterol-rich calf brain powder medium [14]. Later on, several strains have been isolated from feces and intestinal contents of baboons. All these strains, assigned to the genus *Eubacterium*, are no more available in bacterial collections. More recently, a small, anaerobic, gram-positive coccobacillus that reduces cholesterol to coprostanol was isolated from a hog sewage lagoon and named *Eubacterium coprostanoligenes* ATCC 51222 [15]. The mechanism of cholesterol reduction by *Eubacterium coprostanoligenes* was deciphered, showing that isomerization of the 5-6 double bond to a 4-5 double bond occurred via a mechanism involving the transfer of C-4 H to the C-6 position during the cholesterol-to-coprostanol conversion [16]. This indicated an indirect pathway involving the formation of 4-cholesten-3-one. Lately, the first cholesterol-reducing bacterium from human origin has been isolated and characterized [17] (Figure 2).

Unlike all other cholesterol-reducing strains isolated so far, this isolate belongs to the genus *Bacteroides*. More precisely, this strain, that we named *Bacteroides* sp. strain D8, is closely related to *Bacteroides dorei* species. Yet, no cholesterol-reducing activity was detected in cultures of *B. dorei* type strain. As observed with *E. coprostanoligenes*, *Bacteroides* sp. strain D8 starts to reduce cholesterol to coprostanol on the third day of growth *in vitro* and seven days are necessary to achieve complete cholesterol conversion. 4-cholesten-3-one and coprostanone are detected during cholesterol conversion by *Bacteroides* sp. strain D8. Moreover, it was observed that this strain was able to convert 4-cholesten-3-one and coprostanone to coprostanol *in vitro*, suggesting an indirect pathway for coprostanol production by *Bacteroides* sp. strain D8 [17].

Coprostanol, unlike cholesterol, is poorly absorbed by the human intestine [18]. Hence, conversion of cholesterol to coprostanol might be a way of lowering serum cholesterol in humans and thus reducing the risk of cardiovascular disease. Indeed, our recent data suggest a relationship between intestinal microbiota and cholesterol metabolism [19] and it has been demonstrated that germfree conditions accelerates the atherosclerosis in ApoE-deficient mice [20]. In humans, modulation of the gut microbiota by neomycin impacts serum cholesterol and fecal sterols in hypercholesterolemic patients [21] and an inverse relationship has been observed between serum cholesterol levels and the coprostanol to cholesterol ratio in feces [22]. Therefore, several studies with animal models were designed to investigate the effect of feeding of *E. coprostanoligenes* on serum cholesterol concentration. In the first study, it was shown that oral administration of *E. coprostanoligenes* resulted in a significant decrease of plasma cholesterol concentration in dietary induced hypercholesterolemic rabbits. Moreover, this hypocholesterolemic effect lasted for at least 34 days after the last bacterial feeding [23]. Concurrently, coprostanol-to-cholesterol ratio was found significantly greater in the digestive contents of treated rabbits. Nevertheless, oral administration *E. coprostanoligenes* to laying hens and mice failed to affect plasma cholesterol [24,25].

Finally, only a few cholesterol-reducing bacteria have been isolated and the genes or enzymes involved in this metabolism are still unknown. Identification of these genes would be a prerequisite to a possible application for medical purpose. Moreover, the consequences on health of the intestinal cholesterol metabolism by the gut microbiota are currently unexplored. Concurrently, more studies on animal models, especially using gnotobiotic animals, are needed to determine the effect of this bacterial metabolism on plasma cholesterol. Interestingly, it was shown that human microbiota retained its level of cholesterol-reducing bacterial population and cholesterol-reducing activity in gnotobiotic rats [26]. Rodents harboring a human gut microbiota could therefore be used as a model to explore the impact on cholesterolemia and atherosclerosis development of gut microbial cholesterol metabolism and to decipher if a dysbiosis may change host cholesterol metabolism.

## 3. Metabolism of Bile Acids by the Gut Microbiota

Bile acids are saturated, hydroxylated C24 cyclopentanepheznanthrene sterols. Primary bile acids (in humans, cholic and chenodeoxycholic acids are the two primary bile acids) are synthesized from cholesterol in the liver and conjugated to either taurine or glycine via an amide linkage at the C24 carboxyl [27]. They are then excreted through the canaliculi to the biliary system. More than 95% of the bile acids secreted in bile are reabsorbed in the distal ileum and return to the liver [28]. This process is named enterohepatic circulation and four to twelve cycles occur each day. The main function of bile acids is to assist the absorption of dietary lipids and lipid-soluble nutrients. However, they are now recognized as signalling molecules through activation of receptors like farnesoid X receptor (FXR) or G protein-coupled receptor (TGR5). Therefore, they may modulate lipid, glucose, energy and drug metabolisms as well as their own biosynthesis [29]. The part of the bile acids that escape the enterohepatic circulation (200 to 800 mg daily in humans) passes into the colon where they undergo bacterial metabolism. These bacterial conversions appear very early in life as 16 different bile acids were identified in meconium [30]. The main bile salt conversions in the human gut include deconjugation, oxidation and epimerization of hydroxyl groups at C3, C7 and C12, 7-dehydroxylation, esterification and desulfatation (Table 1) [31] and lead to the presence of over 20 different secondary bile acids in adult human feces.

**Table 1:** Bacterial genera of the gut microbiota involved in bile acids metabolism.

Reactions	Bacterial genera
deconjugation	*Bacteroides*, *Bifidobacterium*, *Clostridium*, *Lactobacillus*, *Listeria*
oxidation and epimerization	*Bacteroides*, *Clostridium*, *Escherichia*, *Egghertella*, *Eubacterium*, *Peptostreptococcus*, *Ruminococcus*
7-dehydroxylation	*Clostridium*, *Eubacterium*
esterification	*Bacteroides*, *Eubacterium*, *Lactobacillus*
desulfatation	*Clostridium*, *Fusobacterium*, *Peptococcus*, *Pseudomonas*

### 3.1. Deconjugation

The hydrolysis of the C24 N-acyl amide bond of conjugated bile acids is catalyzed by bile salt hydrolases (BSHs). Most BSHs hydrolyze both glycine and taurine conjugated bile acids whereas a few display strong specificity. BSH genes have been detected in the main bacterial genera of the gut microbiota [32] and the enzyme has been purified from *Bacteroides fragilis*, *B. vulgatus*, *Clostridium perfringens*, *Listeria monocytogenes* and several species of *Lactobacillus* and *Bifidobacterium*. Also, the BSH from *C. perfringens* was crystallized and resolutions at 2.1 and 1.7. A were obtained for the apoenzyme form and for the complex with taurodeoxycholate, respectively [33]. It has been suggested that BSH activity could be a way for bacterial species to detoxify bile acids. Besides, deconjugation was shown to improve the bacterial colonization of the gastrointestinal tract of mammals and deletion of the *bsh* gene significantly reduced infectivity of *L. monocytogenes in vivo* [34]. Lastly, some bacterial species may obtain carbon, nitrogen, and sulfur from bile acids deconjugation. In particular, taurine contains a sulfonic moiety that is reduced to hydrogen sulfide after deconjugation. This metabolism may have health consequences as hydrogen sulfide increases colonocyte turnover and may be associated with inflammatory bowel disease and colon cancer [35].

### 3.2. Oxidation and Epimerization of 3-, 7- and 12- Hydroxy Groups

Bile acid hydroxysteroid dehydrogenases (HSDHs) from intestinal bacteria catalyze the reversible oxidation of hydroxy to oxo groups. Epimerization of hydroxyl groups occurs via stereospecific oxidation followed by stereospecific reduction of the resulting oxo group [36]. Epimerization requires the actions of two stereochemically distinct HSDHs and can be performed by a single species containing both α- and β- HSDHs or by two species, one possessing an α-HSDH and the other a β-HSDH [37]. 3α- and 3β-HSDHs have been detected in the gut microbiota in several bacteria belonging to the Firmicutes phylum whereas intraspecies 3-hydroxy epimerization has been observed only in *Peptostreptococcus productus*, *C. perfringens* and *Eggerthella lenta*. 7α-HSDHs are widespread among members of the genera *Clostridium*, *Eubacterium*, *Bacteroides* or *Escherichia* and 7β-HSDHs have been detected only in Firmicutes. Bacteria capable of intraspecies 7-epimerization include species of the genera *Clostridium*, *Eubacterium*, *Ruminococcus* [37,38]. The genes encoding 7α-HSDHs have been cloned from several species and the crystal structure of the *E. coli* 7α-HSDH has been solved [39]. 12-oxo bile acids are present at very low levels in the feces of healthy humans. 12α- or 12β-HSDHs have been detected in different members of the Firmicutes but to date, no isolate has been found to possess both 12α- and 12β-HSDHs.

### 3.3. 7-Dehydroxylation

Unlike bile acid oxidation and epimerization, dehydroxylation can only occur after deconjugation due to inaccessibility of the hydroxyl group. The 7α-dehydroxylation of the primary bile acids (cholic and chenodeoxycholic acids), leading to deoxycholic and lithocholic acids is the most quantitatively important and the most physiologically significant conversion of bile acids in humans [40]. Deoxycholic acid may therefore account for up to 25% of the total bile acid pool. The known bacterial species which possess 7α-dehydroxylation activity are members of the Firmicutes phylum (*Clostridium*, *Eubacterium*). The bile acid-inducible (bai) enzyme system which dehydroxylates 7α-hydroxy bile acids has been extensively studied in the human intestinal isolate *Clostridium scindens* and *C. hylemonae* [36]. It was first noticed that the induction of 7α-dehydroxylation activity in *C. scindens* by primary bile acids led to the production of new proteins. Accumulation of multiple bile acid intermediates in cell extracts of *C. scindens* induced by cholic acid was then observed suggesting a multistep pathway for this metabolism [41]. Later on, a *bai* regulon, displaying highly conserved gene organization and sequence has been found in *C. scindens* and *C. hylemonae*. Characterization of this operon unravelled the mechanism for bile acid 7α-dehydroxylation in these bacteria [42].

### 3.4. Esterification and Desulfatation

Saponifiable derivatives (esters) of bile acids account for 10 to 30% of the total bile acid content in human feces. Moreover, large amounts of deoxycholic acid oligomers, formed by esterification of the C-24 carboxyl group of one molecule with the 3α-hydroxy group of the next one, are detected in human feces [13]. These esters are not present in bile suggesting their production by intestinal bacteria. Mixed fecal cultures were therefore found to convert bile acids to their C-24 ethyl esters and this activity was detected in a few intestinal isolates belonging to the genera *Bacteroides*, *Eubacterium* and *Lactobacillus* [36]. Bile acids sulfatase activity has been detected in intestinal isolates belonging to the genera *Clostridium*, *Peptococcus*, *Fusobacterium* and *Pseudomonas*. This activity requires a 3α- or 3β-sulfo group (bile acids sulfated at other positions are not desulfated), and a free C24 or C26 carboxyl group. Up to date, enzymes catalyzing the reaction have only been purified from *Pseudomonas testosteroni* [43].

### 3.5. Bile Acids Metabolism in Health and Disease

Recent studies revealed that bile acids exert a much wider range of biological activities than initially recognized [28]. Moreover, it was established that secondary bile acids produced by the gut microbiota are present in peripheral tissues, including liver, kidney and heart, emphasizing their possible broad influence on mammalian homeostasis [44]. Therefore, bile acids metabolism by the gut microbiota may promote health or favour disease development depending on the quantity and type of secondary bile acids produced. As examples, the dehydroxylation of chenodeoxycholic acid lead to lithocholic acid which is toxic to liver cells and has been linked to colon carcinogenesis [45]. Similarly, high levels of deoxycholic acid (known to cause DNA damage) in blood and feces are associated with increased risks of cholesterol gallstone disease and colon and liver cancer [46,47]. Conversely, ursodeoxycholic acid, produced by the epimerization of the 7α-hydroxyl group of chenodeoxycholic acid, is thought to be chemopreventive and is used to treat cholesterol gallstones. Recently, it was also demonstrated that the dysbiosis observed in inflammatory bowel disease lead to decreased bile acids deconjugation and desulfatation activities and then to a modification in the luminal bile acids pool composition which may contribute to chronic inflammation [48]. Therefore, we can see this phenomenon as a vicious circle where a disease lead to a dysbiosis resulting in altered bile acid pool able to worsen the disease state. Interestingly, bile acids may also exert effect on health via an alteration of the gut microbiota, i.e., alterations in host bile acid metabolism associated with disease or diet might cause dysbiosis with health consequences [49]. Hence, it has been demonstrated that an increase in taurocholic acid, due to ingestion of milk fat, stimulates a sulphite-reducing pathobiont, *Bilophila wadsworthia* resulting in development of colitis in genetically susceptible mice that lacked interleukin-10 [50]. Finally, bile acids, gut microbiota and health status are closely linked and influence each other making difficult to comprehend if dysbiosis and altered bile acids pool are a cause or a consequence of the disease.

## 4. Conclusions

Cholesterol and bile acids metabolisms by the gut microbiota have been extensively studied but results have been mainly obtained from classical culturing techniques. Molecular techniques, particularly sequencing of bacterial genomes and of the human gut microbiome, should allow the discovery of novel genes involved in these metabolisms and to understand the real diversity of steroid-converting bacteria. This may help to define the relationship between these bacterial populations and disease risks. Also, colonisation of germfree rodents by steroid-converting bacteria or complex microbiota with different cholesterol and bile acids metabolizing activities should lead to the understanding of the real impact of these metabolisms on host’s physiology as well as the mechanisms involved. Finally, targeting the gut microbiota to modify cholesterol and bile acids metabolisms might be a new preventive or therapeutic approach in various diseases including cholesterol gallstone disease, colon and liver cancers, inflammatory and metabolic diseases.

## References

[1] Palmer C., Bik E.M., DiGiulio D.B., Relman D.A., Brown P.O. (2007). Development of the human infant intestinal microbiota. PLoS Biol..

[2] Lozupone C.A., Stombaugh J.I., Gordon J.I., Jansson J.K., Knight R. (2012). Diversity, stability and resilience of the human gut microbiota. Nature.

[3] O’Hara A.M., Shanahan F. (2006). The gut flora as a forgotten organ. EMBO Rep..

[4] Eckburg P.B., Bik E.M., Bernstein C.N., Purdom E., Dethlefsen L., Sargent M., Gill S.R., Nelson K.E., Relman D.A. (2005). Diversity of the human intestinal microbial flora. Science.

[5] Qin J., Li R., Raes J., Arumugam M., Burgdorf K.S., Manichanh C., Nielsen T., Pons N., Levenez F., Yamada T. (2010). A human gut microbial gene catalogue established by metagenomic sequencing. Nature.

[6] Gérard P. (2011). Le microbiote intestinal: Composition et fonctions. Phytothérapie.

[7] Van der Velde A.E., Brufau G., Groen A.K. (2010). Transintestinal cholesterol efflux. Curr. Opin. Lipidol..

[8] Schoenheimer R. (1931). New contributions in sterol metabolism. Science.

[9] Kellogg T.F. (1974). Steroid balance and tissue cholesterol accumulation in germfree and conventional rats fed diets containing saturated and polyunsaturated fats. J. Lipid Res..

[10] Midtvedt A.C., Midtvedt T. (1993). Conversion of cholesterol to coprostanol by the intestinal microflora during the first two years of human life. J. Pediatr. Gastroenterol. Nutr..

[11] Veiga P., Juste C., Lepercq P., Saunier K., Beguet F., Gérard P. (2005). Correlation between faecal microbial community structure and cholesterol-to-coprostanol conversion in the human gut. FEMS Microbiol. Lett..

[12] Wilkins T.D., Hackman A.S. (1974). Two patterns of neutral steroid conversion in the feces of normal North Americans. Cancer Res..

[13] Macdonald I.A., Bokkenheuser V.D., Winter J., McLernon A.M., Mosbach E.H. (1983). Degradation of steroids in the human gut. J. Lipid Res..

[14] Eyssen H.J., Parmentier G.G., Compernolle F.C., de Pauw G., Piessens-Denef M. (1973). Biohydrogenation of sterols by Eubacterium ATCC 21,408--Nova species. Eur. J. Biochem..

[15] Freier T.A., Beitz D.C., Li L., Hartman P.A. (1994). Characterization of *Eubacterium coprostanoligenes* sp. nov., a cholesterol-reducing anaerobe. Int. J. Syst. Bacteriol..

[16] Ren D., Li L., Schwabacher A.W., Young J.W., Beitz D.C. (1996). Mechanism of cholesterol reduction to coprostanol by *Eubacterium coprostanoligenes* ATCC 51222. Steroids.

[17] Gérard P., Lepercq P., Leclerc M., Gavini F., Raibaud P., Juste C. (2007). *Bacteroides* sp. strain D8, the first cholesterol-reducing bacterium isolated from human feces. Appl. Environ. Microbiol..

[18] Lichtenstein A.H. (1990). Intestinal cholesterol metabolism. Ann. Med..

[19] Rabot S., Membrez M., Bruneau A., Gérard P., Harach T., Moser M., Raymond F., Mansourian R., Chou C.J. (2010). Germ-free C57BL/6J mice are resistant to high-fat-diet-induced insulin resistance and have altered cholesterol metabolism. FASEB J..

[20] Stepankova R., Tonar Z., Bartova J., Nedorost L., Rossman P., Poledne R., Schwarzer M., Tlaskalova-Hogenova H. (2010). Absence of microbiota (germ-free conditions) accelerates the atherosclerosis in ApoE-deficient mice fed standard low cholesterol diet. J. Atheroscler. Thromb..

[21] Miettinen T.A. (1979). Effects of neomycin alone and in combination with cholestyramine on serum cholesterol and fecal steroids in hypercholesterolemic subjects. J. Clin. Investig..

[22] Sekimoto H., Shimada O., Makanishi M., Nakano T., Katayama O. (1983). Interrelationship between serum and fecal sterols. Jpn. J. Med..

[23] Li L., Buhman K.K., Hartman P.A., Beitz D.C. (1995). Hypocholesterolemic effect of *Eubacterium coprostanoligenes* ATCC 51222 in rabbits. Lett. Appl. Microbiol..

[24] Li L., Baumann C.A., Meling D.D., Sell J.L., Beitz D.C. (1996). Effect of orally administered *Eubacterium coprostanoligenes* ATCC 51222 on plasma cholesterol concentration in laying hens. Poult. Sci..

[25] Li L., Batt S.M., Wannemuehler M., Dispirito A., Beitz D.C. (1998). Effect of feeding of a cholesterol-reducing bacterium, *Eubacterium coprostanoligenes*, to germ-free mice. Lab. Anim. Sci..

[26] Gérard P., Beguet F., Lepercq P., Rigottier-Gois L., Rochet V., Andrieux C., Juste C. (2004). Gnotobiotic rats harboring human intestinal microbiota as a model for studying cholesterol-to-coprostanol conversion. FEMS Microbiol. Ecol..

[27] Hofmann A.F., Hagey L.R., Krasowski M.D. (2010). Bile salts of vertebrates: Structural variation and possible evolutionary significance. J. Lipid Res..

[28] Zwicker B.L., Agellon L.B. (2013). Transport and biological activities of bile acids. Int. J. Biochem. Cell Biol..

[29] Hylemon P.B., Zhou H., Pandak W.M., Ren S., Gil G., Dent P. (2009). Bile acids as regulatory molecules. J. Lipid Res..

[30] Jonsson G., Midtvedt A.C., Norman A., Midtvedt T. (1995). Intestinal microbial bile acid transformation in healthy infants. J. Pediatr. Gastroenterol. Nutr..

[31] Midtvedt T. (1974). Microbial bile acid transformation. Am. J. Clin. Nutr..

[32] Jones B.V., Begley M., Hill C., Gahan C.G., Marchesi J.R. (2008). Functional and comparative metagenomic analysis of bile salt hydrolase activity in the human gut microbiome. Proc. Natl. Acad. Sci. USA.

[33] Rossocha M., Schultz-Heienbrok R., von Moeller H., Coleman J.P., Saenger W. (2005). Conjugated bile acid hydrolase is a tetrameric N-terminal thiol hydrolase with specific recognition of its cholyl but not of its tauryl product. Biochemistry.

[34] Dussurget O., Cabanes D., Dehoux P., Lecuit M., Buchrieser C., Glaser P., Cossart P. (2002). Listeria monocytogenes bile salt hydrolase is a PrfA-regulated virulence factor involved in the intestinal and hepatic phases of listeriosis. Mol. Microbiol..

[35] Carbonero F., Benefiel A.C., Alizadeh-Ghamsari A.H., Gaskins H.R. (2012). Microbial pathways in colonic sulfur metabolism and links with health and disease. Front. Physiol..

[36] Ridlon J.M., Kang D.J., Hylemon P.B. (2006). Bile salt biotransformations by human intestinal bacteria. J. Lipid Res..

[37] Lepercq P., Gérard P., Béguet F., Raibaud P., Grill J.P., Relano P., Cayuela C., Juste C. (2004). Epimerization of chenodeoxycholic acid to ursodeoxycholic acid by Clostridium baratii isolated from human feces. FEMS Microbiol. Lett..

[38] Lepercq P., Gérard P., Béguet F., Grill J.-P., Relano P., Cayuela C., Juste C. (2004). Isolates from normal human intestinal flora but not lactic acid bacteria exhibit 7alpha- and 7beta-hydroxysteroid dehydrogenase activities. Microb. Ecol. Health Dis..

[39] Tanaka N., Nonaka T., Tanabe T., Yoshimoto T., Tsuru D., Mitsui Y. (1996). Crystal structures of the binary and ternary complexes of 7 alpha-hydroxysteroid dehydrogenase from *Escherichia* coli. Biochemistry.

[40] Hamilton J.P., Xie G., Raufman J.P., Hogan S., Griffin T.L., Packard C.A., Chatfield D.A., Hagey L.R., Steinbach J.H., Hofmann A.F. (2007). Human cecal bile acids: Concentration and spectrum. Am. J. Physiol. Gastrointest. Liver Physiol..

[41] Hylemon P.B., Melone P.D., Franklund C.V., Lund E., Bjorkhem I. (1991). Mechanism of intestinal 7 alpha-dehydroxylation of cholic acid: Evidence that allo-deoxycholic acid is an inducible side-product. J. Lipid Res..

[42] Ridlon J.M., Kang D.J., Hylemon P.B. (2010). Isolation and characterization of a bile acid inducible 7alpha-dehydroxylating operon in Clostridium hylemonae TN271. Anaerobe.

[43] Tazuke Y., Matsuda K., Adachi K., Tsukada Y. (1998). Purification and properties of a novel sulfatase from Pseudomonas testosteroni that hydrolyzed 3 beta-hydroxy-5-cholenoic acid 3-sulfate. Biosci., Biotechnol., Biochem..

[44] Swann J.R., Want E.J., Geier F.M., Spagou K., Wilson I.D., Sidaway J.E., Nicholson J.K., Holmes E. (2011). Systemic gut microbial modulation of bile acid metabolism in host tissue compartments. Proc. Natl. Acad. Sci. USA.

[45] Hofmann A.F. (2004). Detoxification of lithocholic acid, a toxic bile acid: Relevance to drug hepatotoxicity. Drug Metab. Rev..

[46] McGarr S.E., Ridlon J.M., Hylemon P.B. (2005). Diet, anaerobic bacterial metabolism, and colon cancer: A review of the literature. J. Clin. Gastroenterol..

[47] Yoshimoto S., Loo T.M., Atarashi K., Kanda H., Sato S., Oyadomari S., Iwakura Y., Oshima K., Morita H., Hattori M. (2013). Obesity-induced gut microbial metabolite promotes liver cancer through senescence secretome. Nature.

[48] Duboc H., Rajca S., Rainteau D., Benarous D., Maubert M.A., Quervain E., Thomas G., Barbu V., Humbert L., Despras G. (2013). Connecting dysbiosis, bile-acid dysmetabolism and gut inflammation in inflammatory bowel diseases. Gut.

[49] Trauner M., Fickert P., Tilg H. (2013). Bile acids as modulators of gut microbiota linking dietary habits and inflammatory bowel disease: A potentially dangerous liaison. Gastroenterology.

[50] Devkota S., Wang Y., Musch M.W., Leone V., Fehlner-Peach H., Nadimpalli A., Antonopoulos D.A., Jabri B., Chang E.B. (2012). Dietary-fat-induced taurocholic acid promotes pathobiont expansion and colitis in Il10^−/−^ mice. Nature.

